# Cerebral Venous Sinus Thrombosis Following Nitrous Oxide Abuse: A Clinical Reminder of a Growing Trend

**DOI:** 10.7759/cureus.92570

**Published:** 2025-09-17

**Authors:** Madison Kober, Sahil Sardana, Maryam Zulfiqar, Tyler Krug, Taranjit Gill

**Affiliations:** 1 Neurology, Allegheny Health Network, Pittsburgh, USA; 2 Vascular Neurology, Allegheny Health Network, Pittsburgh, USA; 3 Medicine, Allegheny Health Network, Pittsburgh, USA

**Keywords:** cerebral venous sinus thrombosis (cvst), hypercoaugulable, hyperhomocysteinemia (hhcy), nitrous oxide abuse, prothrombotic state, stroke, stroke in young adult, substance-induced thrombosis, vitamin b12 levels, whippets

## Abstract

Cerebral venous sinus thrombosis (CVST) is a rare cause of stroke that may mimic other intracranial disorders. A 32-year-old male with bipolar disorder and prior venous thromboembolism presented with severe occipital headache, progressive blurred vision, and a brief sensory episode. He reported recent chronic nitrous oxide use. Imaging revealed extensive thrombosis of the superior sagittal, bilateral transverse, and sigmoid sinuses with venous congestion but no infarction. Laboratory studies showed markedly elevated homocysteine, elevated methylmalonic acid, and low vitamin B12 levels. He was treated with anticoagulation, parenteral vitamin B12, folic acid, and acetazolamide for suspected intracranial hypertension. Symptoms improved, and he was discharged for continued rehabilitation. This case underscores nitrous oxide-induced functional vitamin B12 deficiency and hyperhomocysteinemia as potential contributors to CVST, especially in younger patients without conventional risk factors. Early recognition, targeted metabolic correction, and cessation of exposure are essential to prevent complications and support recovery.

## Introduction

Cerebral venous sinus thrombosis (CVST) is a rare but serious form of stroke caused by thrombosis within the dural venous sinuses or cerebral veins, accounting for less than 1% of all strokes. Nitrous oxide (N₂O) abuse has emerged as an increasingly recognized cause of thromboembolic events, including CVST. Presentations can range from headache and visual disturbances to seizures and focal neurological deficits [1]. The overlap of symptoms with conditions such as idiopathic intracranial hypertension (IIH) can further complicate the diagnosis, particularly in patients presenting with papilledema and elevated intracranial pressure [1-4].

One underrecognized risk factor for CVST is vitamin B12 deficiency [5]. Chronic recreational use of N₂O, which is increasingly common among young adults, leads to inactivation of vitamin B12 and can result in both neurologic and thrombotic complications [6-8].

We present a case of CVST in a young adult with a history of chronic nitrous oxide use. This report underscores the importance of evaluating for metabolic and substance-induced etiologies in patients with CVST, particularly when conventional risk factors are absent.

## Case presentation

A 32-year-old male with a history of bipolar disorder, prior deep venous thrombosis (DVT) and pulmonary embolism (PE) attributed to nitrous oxide (N₂O) abuse, and recent completion of a substance rehabilitation program presented for evaluation of suspected bilateral subdural hematomas.

The patient reported last using N₂O approximately 11 days prior to admission. Seven days before the presentation, he developed a right-sided occipital headache rated 9/10 in intensity, most severe in the mornings with gradual improvement throughout the day. He also experienced progressively worsening blurred vision. On the day of admission, he noted a transient 12-minute episode of numbness affecting the left jaw and left hand.

Initial laboratory evaluation at an outside hospital revealed leukocytosis (WBC 16,000/µL), hemoglobin of 17.4 g/dL, and a positive urine drug screen for amphetamines, later attributed to a prescribed dose of lisdexamfetamine. Urinalysis was unremarkable (Table 1). 

**Table 1:** Laboratory findings (pertinent) demonstrating nitrous oxide–induced functional vitamin B12 deficiency and hyperhomocysteinemia. The thrombophilia panel, including protein C, protein S, antithrombin III, Factor V Leiden, prothrombin mutation, and antiphospholipid antibodies, was unremarkable and is not shown.

Test	Patient Value	Reference Range	Interpretation
White Blood Cell Count (WBC)	16,000 /µL	4,000 – 11,000 /µL	Elevated
Hemoglobin (Hb)	17.4 g/dL	13.5 – 17.5 g/dL (M)	Upper-normal
Homocysteine	64.6 µmol/L	5 – 15 µmol/L	Markedly elevated
Methylmalonic Acid (MMA)	1053 nmol/L	70 – 270 nmol/L	Markedly elevated
Vitamin B12	159 pg/mL	200 – 900 pg/mL	Low
Urine Drug Screen	Positive for amphetamines (due to Lisdexamfetamine)	Negative	Drug precribed/Iatrogenic

**Figure 1:**
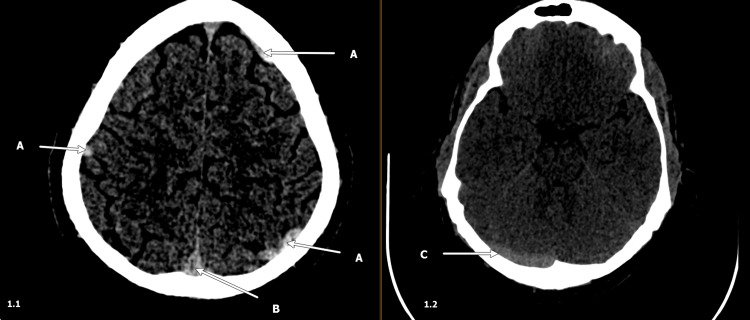
Hyperdense vein signs on non-contrast CT Head. (A) Cortical veins demonstrating hyperdensity due to thrombus. (B) Superior sagittal sinus with hyperdensity. (C) Right transverse sinus demonstrating cord-like hyperattenuation (Cord sign). CT: Computed Tomography

**Figure 2:**
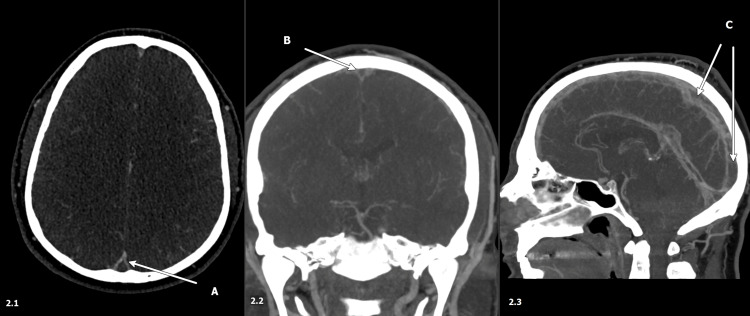
Superior sagittal sinus thrombosis on CTV. (A) Axial section demonstrating empty delta sign. (B) Coronal section demonstrating empty delta sign. (C) Sagittal section showing filling defect. CTV: Computed Tomography Venogram

Non-contrast CT of the head (Figure 1) demonstrated increased density in the cortical veins and dural venous sinuses without evidence of hemorrhage. CT angiography of the head and neck showed no arterial stenosis or occlusion, but CT venography revealed extensive CVST involving the superior sagittal sinus, bilateral transverse and sigmoid sinuses, and the right jugular bulb (Figure 2). An MRI of the brain without contrast showed signs of venous congestion/stasis but no strokes.

Neurological examination was non-focal with a National Institutes of Health Stroke Scale (NIHSS) score of 0. The patient denied alcohol or other illicit drug use and was a non-smoker. His modified Rankin Scale score on admission was 0.

A hypercoagulable workup revealed markedly elevated homocysteine (64.6 µmol/L), elevated methylmalonic acid (1053 nmol/L), and severely reduced vitamin B12 (159 pg/mL). Other thrombophilia tests were unremarkable. These findings were attributed to chronic nitrous oxide abuse, with subsequent functional cobalamin deficiency and hyperhomocysteinemia contributing to CVST. He was started on a heparin infusion protocol for acute thrombus management.

**Figure 3:**
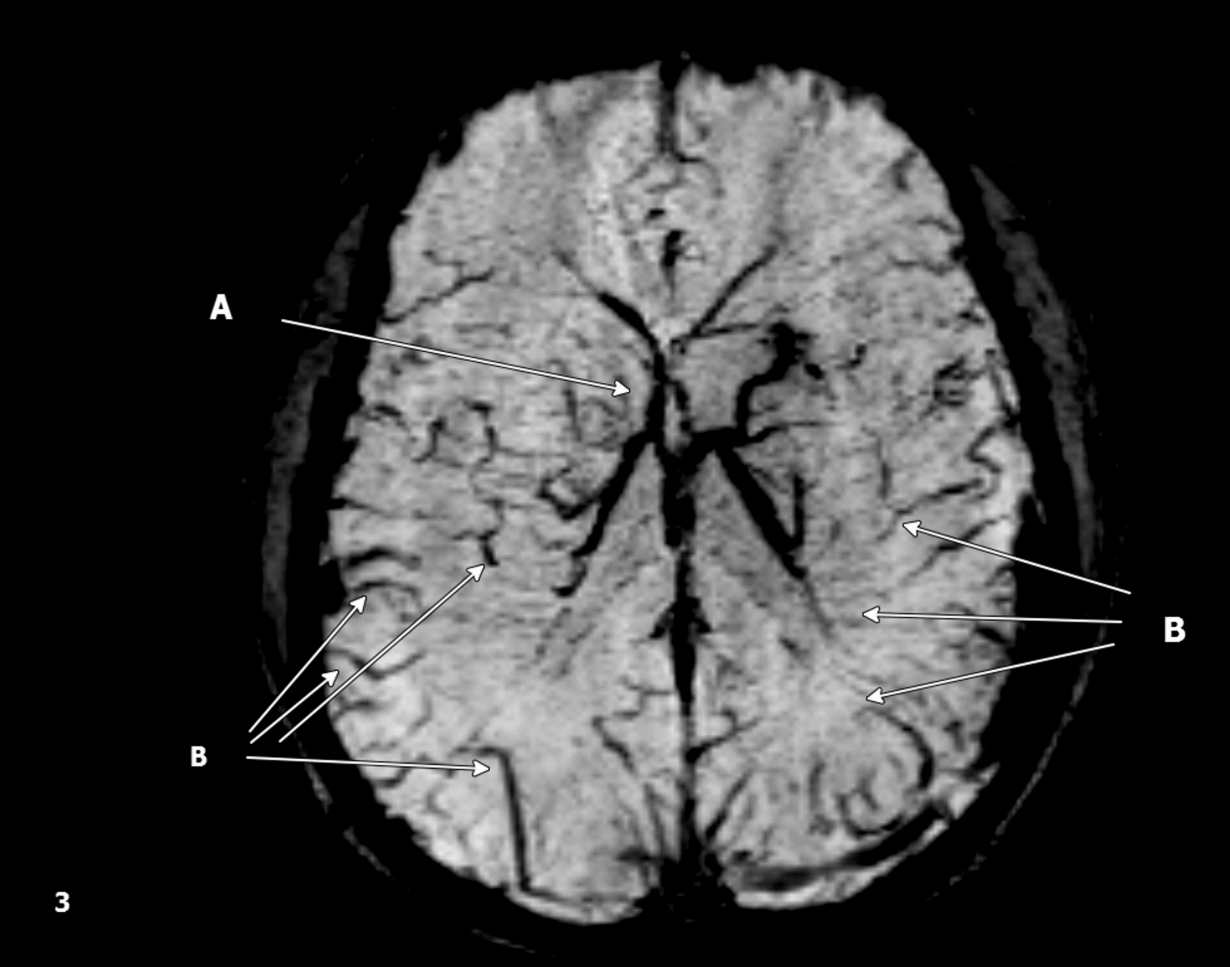
Axial SWI MRI sequence findings suggestive of venous congestion: (A) lateral ventricles with radiating linear hypointense SWI foci and (B) cerebral sulci and white matter with linear SWI signal loss and faint enhancement, likely venous congestion/stasis. SWI: Susceptibility Weighted Imaging, MRI: Magnetic Resonance Imaging

During hospitalization, the patient developed positional headaches and persistent bilateral blurred vision, which worsened while lying down. Acetazolamide 250 mg twice daily was initiated for presumed intracranial hypertension. An ophthalmology consultation was obtained, and outpatient follow-up was recommended. Brain MRI (Figure 3) confirmed venous congestion and stasis with prominent medullary venous collaterals, but no evidence of venous infarction.

Heparin was later transitioned to apixaban 10 mg twice daily for seven days, followed by 5 mg twice daily for a planned six-month course. He also received intramuscular vitamin B12 replacement and folic acid supplementation. His headache was managed conservatively with acetaminophen.

The patient remained clinically stable throughout his hospitalization, with gradual improvement in symptoms. He was discharged in stable condition back to his rehabilitation center to continue substance use recovery. Outpatient neurology and primary care follow-up were arranged for continued management of CVST and nutritional repletion.

## Discussion

N₂O abuse has been increasingly recognized as a significant cause of thromboembolic events, including CVST. The recreational use of nitrous oxide, commonly referred to as "whippets," can result in substantial neurological and thrombotic complications, particularly in young adults. The underlying pathophysiology involves the oxidation and inactivation of vitamin B12 (cobalamin), which is essential for key metabolic pathways, including the methylmalonic acid and homocysteine cycles [6]. Vitamin B12 deficiency disrupts methionine synthase activity, leading to the accumulation of homocysteine, a well-established prothrombotic factor. Elevated homocysteine levels impair endothelial function and interfere with the natural anticoagulant mechanisms, specifically by inhibiting thrombomodulin and antithrombin III activity, as well as promoting tissue factor expression on endothelial cells. These alterations collectively foster a prothrombotic state, particularly pronounced in the low-shear venous circulation, where the balance between coagulation and anticoagulation is more easily disturbed [1,2].

Homocysteine contributes to thrombogenesis through multiple mechanisms. It induces oxidative stress and endothelial injury, which in turn compromises nitric oxide (NO) production, an important vasodilator and inhibitor of platelet aggregation [5]. Additionally, homocysteine enhances the expression of procoagulant factors such as tissue factors and reduces the activity of anticoagulant pathways, including protein C and antithrombin III, further tipping the hemostatic balance towards thrombosis. Elevated homocysteine also increases platelet aggregation, amplifying thrombus formation [6,7].

Clinically, nitrous oxide abuse has been associated not only with DVT and PE but also with CVST, as exemplified by this case. Moreover, neurological sequelae, including polyneuropathy, myelopathy, encephalopathy, and subacute combined degeneration, have been documented [8].

The cornerstone of management involves cessation of nitrous oxide exposure and replenishment of vitamin B12 stores, typically through parenteral administration of hydroxocobalamin or cyanocobalamin. Treatment regimens align with those used in other causes of vitamin B12 deficiency, commonly consisting of 1 mg intramuscular injections every other day for 1-2 weeks, followed by weekly doses until clinical improvement is observed, and then monthly for maintenance. Oral supplementation is generally inadequate in the setting of ongoing nitrous oxide exposure due to persistent functional inactivation despite normal or elevated serum B12 levels [9].

Patients with severe neurological impairments may require multidisciplinary care, including rehabilitation and addiction medicine support. Despite treatment, long-term complications of recreational nitrous oxide use may persist and include sensory deficits such as paresthesias, chronic headaches, mood disorders (depression and anxiety), and cognitive impairments [1].

## Conclusions

Vitamin B12 deficiency secondary to chronic nitrous oxide abuse results in functional inactivation of methionine synthase, culminating in elevated homocysteine levels and the establishment of a hypercoagulable state. This biochemical disruption significantly increases the risk of venous thromboembolism, including cerebral venous sinus thrombosis. Timely recognition of this mechanism is critical, particularly in young patients presenting with thrombotic events and a history of recreational nitrous oxide use. Early identification and appropriate management, including vitamin B12 repletion, anticoagulation, and cessation of nitrous oxide exposure, are essential to prevent further complications and improve clinical outcomes.

## References

[1] Saposnik G, Bushnell C, Coutinho JM (2024). Diagnosis and management of cerebral venous thrombosis: a scientific statement from the American Heart Association. Stroke.

[2] Xie JS, Donaldson L, Margolin E (2022). Papilledema: A review of etiology, pathophysiology, diagnosis, and management. Surv Ophthalmol.

[3] Ducros A, Biousse V (2015). Headache arising from idiopathic changes in CSF pressure. Lancet Neurol.

[4] Colman BD, Boonstra F, Nguyen MN (2024). Understanding the pathophysiology of idiopathic intracranial hypertension (IIH): a review of recent developments. J Neurol Neurosurg Psychiatry.

[5] Herrmann W (2001). The importance of hyperhomocysteinemia as a risk factor for diseases: an overview. Clin Chem Lab Med.

[6] Joncquel Chevalier-Curt M, Grzych G, Tard C (2022). Nitrous oxide abuse in the emergency practice, and Review of toxicity mechanisms and potential markers. Food Chem Toxicol.

[7] Caris MG, Kuipers RS, Kiestra BE (2023). Nitrous oxide abuse leading to extreme homocysteine levels and thrombosis in young adults: a case series. J Thromb Haemost.

[8] Temple C, Horowitz BZ (2022). Nitrous oxide abuse induced subacute combined degeneration despite patient initiated B12 supplementation. Clin Toxicol (Phila).

[9] Pugliese RS, Slagle EJ, Oettinger GR (2015). Subacute combined degeneration of the spinal cord in a patient abusing nitrous oxide and self-medicating with cyanocobalamin. Am J Health Syst Pharm.

